# CAREGIVER HEALTH LITERACY AS A MODIFIABLE TARGET TO PROMOTE OLDER ADULT HEALTH

**DOI:** 10.1093/geroni/igac059.015

**Published:** 2022-12-20

**Authors:** Rachel O'Conor, Morgan Eifler, Lauren Opsasnick, Laura Curtis, Julia Yoshino Benavente, Lee Lindquist, Michael Wolf

**Affiliations:** Northwestern University, Chicago, Illinois, United States; Northwestern University, Chicago, Illinois, United States; Northwestern University, Chicago, Illinois, United States; Northwestern University, Chicago, Illinois, United States; Northwestern University, Chicago, Illinois, United States; Northwestern University, Chicago, Illinois, United States; Northwestern University, Chicago, Illinois, United States

## Abstract

Many older adults receive assistance in managing chronic conditions. Yet complicating the utility of caregiver support is whether caregivers have sufficient skills to aid in a patient’s self-care. Health literacy (HL) is as an important determinant of older adults’ health outcomes, but few studies have examined caregiver HL and patient outcomes. We interviewed 162 patient-caregiver dyads during an ongoing cognitive aging cohort study to examine associations between caregiver HL, measured using the Newest Vital Sign, and older adults’ health outcomes. Physical function and mental health symptoms were assessed using PROMIS short form assessments. Patients’ also self-reported emergency department (ED) visits and hospitalizations over the past 12 months. Chi-square and t-tests were performed, as appropriate. Patients were on average 73 years old and managing 4 comorbidities. The majority were female (70%), identified as Black (35%) or White (60%). Caregivers’ mean age was 64 years; half were female (56%) and had limited HL (48%). Limited caregiver HL was associated with poorer physical function (M=43.0 (8.5) vs. M=46.0 (9.1), p=0.05), greater comorbidities (M=4.0 (1.9) vs M=3.3 (1.8), p=0.02) and more ED visits in the past year (36.7% vs. 19.3%, p=0.01). No differences by caregiver HL were observed for patients’ mental health or hospitalization. Findings suggest that caregivers with limited HL are caring for medically complex patients, and further research should examine whether limited caregiver HL leads to poorer self-management of chronic conditions. The development of HL training for caregivers may better equip them to assist older adults and improve older adult health.

